# Injury-elicited stressors alter endogenous retrovirus expression in lymphocytes depending on cell type and source lymphoid organ

**DOI:** 10.1186/1471-2172-14-2

**Published:** 2013-01-05

**Authors:** Kang-Hoon Lee, Debora Lim, Tajia Green, David Greenhalgh, Kiho Cho

**Affiliations:** 1 Department of Surgery, Burn Research, Shriners Hospitals for Children Northern California, University of California, Davis, Sacramento, CA, 95817, USA

**Keywords:** B-cell, T-cell, Burn, Stress, Murine leukemia virus-type endogenous retrovirus

## Abstract

**Background:**

Murine leukemia virus-type endogenous retroviruses (MuLV-ERVs) constitute ~10% of the mouse genome and are associated with various pathophysiologic processes. In this study, we examined whether MuLV-ERVs’ response to burn-elicited stressors is specific for certain lymphocyte populations and/or locations of lymphoid organ.

**Results:**

B- and T-cells, which were sorted from nine lymphoid organs of C57BL/6J mice after burn, were subjected to MuLV-ERV expression analyses. Overall, the post-burn MuLV-ERV expression pattern was dependent on lymphocyte type, time after injury, location of lymphoid organ, and MuLV-ERV type. For instance, the MuLV-ERV expression in T-cells from the thymus and three cervical lymph nodes decreased at 3 hours post-burn while the expression of some MuLV-ERVs was augmented in B-cells derived from the mesenteric lymph node. The MuLV-ERV U3 sequences population of the burn-24 hours group was less diverse in comparison to the no burn and burn-3 hours groups. In addition, it was apparent that at the 24 hours time point, the U3 populations of B-cells from both no burn and burn groups were less heterogeneous than the T-cells’ U3 populations. Using the U3 sequences, some of which were isolated only from specific experimental groups (B- vs. T-cells; no burn vs. burn), as probes, 51 putative MuLV-ERVs, including 16 full-length proviruses, were mapped followed by characterization of their biologic properties.

**Conclusion:**

MuLV-ERVs’ response to burn-elicited stressors may be differentially controlled depending on lymphocyte type, location of lymphoid organ, MuLV-ERV type, and stress duration.

## Background

Proviral sequences of the murine leukemia virus-type endogenous retroviruses (MuLV-ERVs) are genomic footprints reflecting the ancient infection of germ-line cells and are presumed to constitute ~10% of the mouse genome [1]. Although most MuLV-ERV genomes are reported to be defective primarily due to mutation and recombination events, some retain intact proviral genome features, such as identical long terminal repeats (LTRs) at both ends, which are formed by retroviruses’ unique replication process, and coding potential for the major structural genes (*gag, pol* and *env*) which are essential for virion assembly [2-4]. Expression of MuLV-ERVs is mostly controlled by their U3 promoters located on the 5^′^ LTR, in conjunction with the transcription machinery that is dynamically and uniquely formed within individual cells [5].

Recent studies provide evidence that human endogenous retroviruses (HERVs) are involved in normal physiology as well as various disease processes [6]. The HERV-W envelope protein, called syncytin, is highly expressed in the syncytiotrophoblast layer and plays a critical role in placental differentiation [7]. In addition, the inflammatory properties of syncytin contribute to the degeneration of oligodendrocytes responsible for the development of multiple sclerosis, an autoimmune disease [8].

Patients with severe burn injury often succumb to systemic immune disorder in conjunction with internal organ damage [9]. However, the mechanisms underlying the post-burn pathologic changes have not yet been fully characterized. The results from recent studies suggest that post-burn systemic inflammatory response is associated with the activation and/or depletion of specific lymphocyte subpopulations, such as CD4^+^CD25^+^ T regulatory cells, lymphocytes expressing CD25, CD69 and CD71, NK-T-cells, and CD8^+^ T-cells [10-12]. Sepsis-elicited stressors induce apoptosis in the spleen, resulting in the depletion of B-cells and accompanying immune malfunction [13]. However, the central signaling molecules responsible for the post-burn activation and/or depletion of lymphocytes remain to be elucidated.

Our recent studies demonstrated that burn-elicited stressors differentially alter the expression of MuLV-ERVs in a range of internal organs, including various lymphoid tissues, in a ERV type- and organ-specific manner [14]. Moreover, some MuLV-ERV envelope proteins were able to modulate the mRNA expression of inflammatory cytokines such as IL-6 and IL-1β in macrophage cells [15]. In this study, we hypothesize that the differential post-burn alterations in MuLV-ERV expression, depending on lymphocyte types and/or lymphoid organs, may be associated with systemic pathogeneses, including immune disorder. We examined whether MuLV-ERVs’ response to burn-elicited stressors is specific depending on lymphocyte type and the location of lymphoid organ followed by an investigation of the biologic characteristics of the relevant MuLV-ERVs.

## Methods

### Animal experiments

The experimental protocols involving mice were approved by the Animal Use and Care Administrative Advisory Committee of the University of California, Davis. Female C57BL/6J mice (12 weeks old) were purchased from Jackson Laboratory-West (Sacramento, CA). The baseline MuLV-ERV expression patterns were examined in nine lymphoid organs (spleen, thymus, and seven lymph nodes [axillary, inguinal, mesenteric and pooled cervicals {mandibular, accessory mandibular, superficial parotid, and deep cervical}]). Three mice were sacrificed for tissue collection by cervical dislocation without any treatment. The burn protocol has been described previously [16]. Briefly, under general anesthesia, an ~18% total body surface area flame burn was generated on the shaved back of mice followed by resuscitation with saline and buprenorphine. Control (no burn) mice were treated the same as burn mice but without the flame burn. The three mice from each experimental group were sacrificed by CO_2_ inhalation at 3 hours and 24 hours after burn followed by the collection of the same nine lymphoid organs as above without any pooling. The burn experiment was repeated four times.

### Cell sorting

The collected tissues were minced, suspended in a trypsin/EDTA solution, and then passed through a cell strainer (BD Biosciences, CA). The cells were washed with PBS containing 0.1% BSA followed by centrifugation at 1,000 *×g* for 5 min at 4°C. Following the removal of red blood cells, cells were sorted into B- and T-cells using pan-B and pan-T Dynabeads (Invitrogen, Carlsbad, CA), respectively, according to the protocols provided by the manufacturer.

### Total RNA isolation and RT-PCR

Total RNAs were isolated from the sorted cells using an RNeasy Mini Kit (Qiagen, Valencia, CA) following homogenization using a QIAshredder (Qiagen). cDNAs were synthesized using Sensiscript reverse transcriptase (Qiagen), RNase inhibitor (Promega, Madison, WI) and an oligo-dT primer (5^′^-GGC CAC GCG TCG ACT AGT ACT TTT TTT TTT TTT TTT T- 3^′^). A set of primers, ERV-U1 (5^′^-CGG GCG ACT CAG TCT ATC GG-3^′^) and ERV-U2 (5^′^-CAG TAT CAC CAA CTC AAA TC-3^′^), was used to amplify the MuLV-ERV U3 regions [17]. β-actin, serving as a normalization control, was amplified with the primer set: 5^′^-CCA ACT GGG ACG ACA TGG AA-3^′^ and 5^′^-GTA GAT GGG CAC AGT GTG GG-3^′^.

### Measurement of relative purity of sorted cell populations

The relative purity of the sorted cell populations was measured by real-time RT-PCR using CD20 (B-cell antigen) primers (5^′^-AAA ACC TCC AGG AAG AGT TTG GTC-3^′^ and 5^′^-CGA TCT CAT TTT CCA CTG GCA AG-3^′^) for B-cells and Thy1 (Thymus cell antigen-1) primers (5^′^-TCC TGC TCT CAG TCT TGC-3^′^ and 5^′^-AGT AGT CGC CCT CAT CCT TG-3^′^) for T-cells. Real-time RT-PCR was performed using the Brilliant-III SYBR Green QPCR kit from Agilent Technologies (La Jolla, CA) and the results were analyzed using the MxPro (v.4.10) program (Agilent Technologies). Cell type specific mRNA levels (CD20 and Thy1) were calculated from each sorted cell population with the level of β-actin as a normalization control, using the same primer set as above. The relative purity of each cell population was calculated with the assumption that there are only B- and T-cells in each sorted cell population and CD20 and Thy1 are exclusively expressed in B-cells and T-cells, respectively.

### Densitometry and statistical analysis

The density of the PCR products was measured using KODAK Molecular Imaging Software Version 4.5.1 (Eastman Kodak Company, Rochester, NY). All optical densities were measured based on mass values with the Hi-Lo™ DNA marker (a gift from Minnesota Molecular Inc., Minneapolis, MN). The density of each MuLV-ERV U3 band was normalized to its corresponding β-actin value to obtain the relative intensity of each U3 band. Differences in the relative intensity of the matching MuLV-ERV U3 bands between no burn and burn were calculated as fold changes. Statistical significance (p < 0.05) of fold changes between the matching pairs (no burn and burn) of the individual U3 bands was determined using the data from four burn experiments and Student’s *t*-test.

### Cloning and sequencing

Eighty-eight MuLV-ERV U3 PCR amplicons/bands, which had contrasting densities between individual no burn and burn pairs, were selected for sequence analyses. The PCR amplicons were purified using the QIAquick Gel Extraction kit (Qiagen) and then cloned into the pGEM®-T Easy vector (Promega). Three clones were prepared from each U3 PCR amplicon/band. The plasmid DNAs were prepared using a QIAprep Spin Miniprep kit (Qiagen) for sequencing analysis. Sequencing was performed at a core sequencing facility at the University of California, Davis.

### Alignment and phylogenetic analyses

From a total of 260 U3 sequences, 100 unique U3 sequences were identified using Seqman Pro Version 8.1.2 (DNASTAR, Madison, WI). Multiple sequence alignment of the 100 unique U3s was performed using the Clustal W method in MegAlign (DNASTAR), and a phylogenetic tree was generated by the HYPERTREE program [18]. Seven U3 sequences, representing the seven phylogenetic branches, were subjected to a multiple alignment analysis to examine the U3 sequence features of direct repeats (1/1*, 4/4*, 5/5*, and 6/6*), insertion of 190 nucleotides, and unique region (2) based on the protocol by Tomonaga *et al.*[17,19].

### Profiling of transcription regulatory elements

The 100 unique MuLV-ERV U3 sequences were surveyed for putative transcription regulatory elements using the MatInspector program (Genomatix, Munich, Germany) within the vertebrate matrix group with a core similarity higher than 0.95 [20].

### Determination of population diversity of U3 sequences within each experimental group

Using the MEGA4 program [21], the population diversity of U3 sequences was measured in each experimental group and compared among different groups in two analyses: a) no burn, burn-3 hours, and burn-24 hours, and b) no burn-B-cells, burn-24 hours-B-cells, no burn-T-cells, and burn-24 hours-T-cells.

### *In silico* mapping of putative MuLV-ERVs and characterization of their biological properties

The C57BL/6J mouse genome database from the National Center for Biotechnology Information (NCBI) was surveyed for putative MuLV-ERVs with a greater than 98% identity using the 100 unique U3 sequences as mining probes and the Advanced BLAST program (NCBI). The coding potential of each MuLV-ERV was determined using the Vector NTI program (Invitrogen). Three polypeptide genes (*gag, pol*, and *env*) were noted as intact (+), partial (P), or defective (−) relative to the references (AY219567.2 and AF033811). Each MuLV-ERV was mapped with the chromosomal location and strand orientation. The primer binding site (PBS) of each MuLV-ERV was identified by examination of a stretch of 18 nucleotides immediately downstream of the 5^′^ LTR region [22]. In addition, the annotated gene(s) within 100 Kb upstream and downstream of each MuLV-ERV locus, if any, were mapped.

## Results and discussion

### Lymphocyte type-specific MuLV-ERV expression patterns in lymphoid organs of normal mice

**Figure 1:**
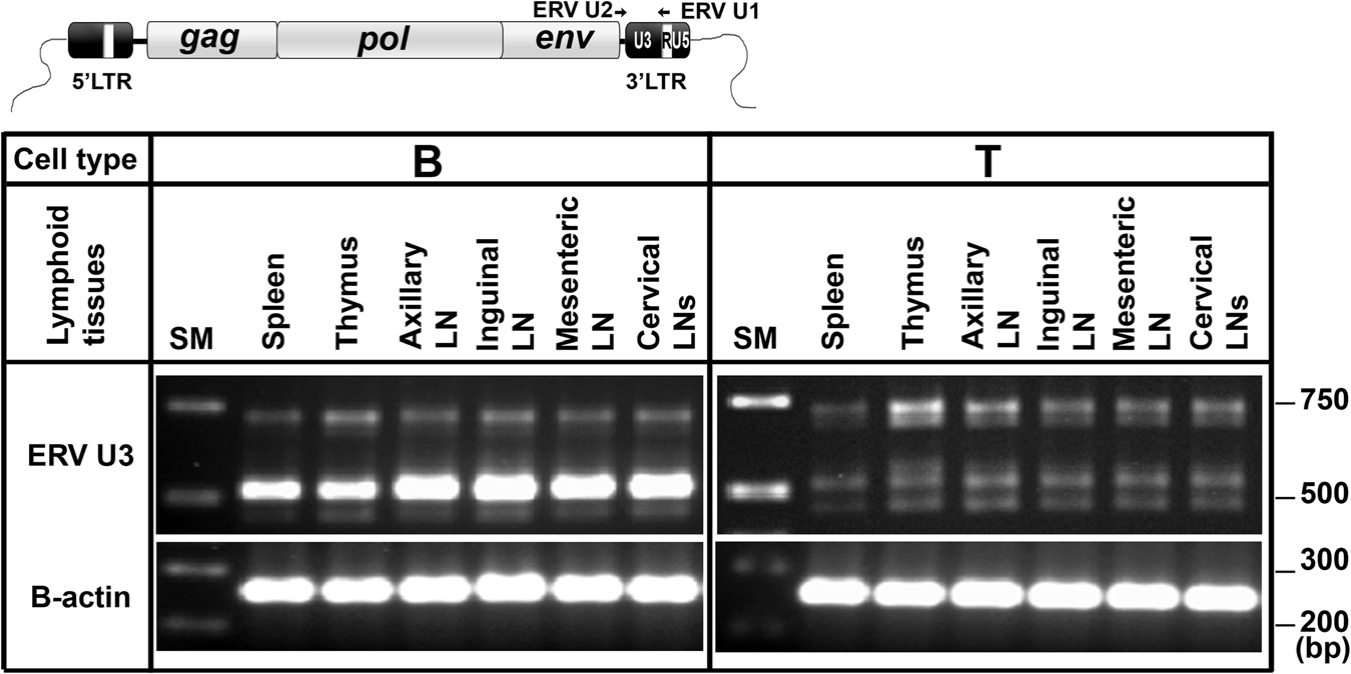
**MuLV-ERV expression in B- and T-cells derived from lymphoid organs of C57BL/6J mice.** MuLV-ERV expression was examined in B- and T-cells isolated from the spleen, thymus, and seven different lymph nodes of female C57BL/6J mice without any treatment. A schematic drawing of a typical MuLV-ERV proviral structure indicates the relative locations of a set of primers used for amplification of the U3 regions. Differential MuLV-ERV expression patterns were observed in B- and T-cells derived from various lymphoid organs. LN (lymph node), SM (size marker).

Prior to the investigation of post-burn alterations in MuLV-ERV expression in B- and T-cells of different lymphoid organs (spleen, thymus, axillary lymph node [LN], inguinal LN, mesenteric LN, and cervical LNs), baseline MuLV-ERV expression levels were examined in B- and T-cells isolated from ~12 week old female C57BL/6J mice. The relative purity of the sorted cell populations was determined by measuring CD20 (for B-cells) and Thy1 (for T-cells) levels by real-time RT-PCR. The relative purity of the sorted B- and T-cell populations was greater than 93%, except for the B-cell population in the thymus (~65%). Throughout the lymphoid organs examined, the MuLV-ERV expression pattern, which was examined by RT-PCR amplification of the 3^′^ U3 regions, was substantially different between B- and T-cells (Figure 1). The expression pattern of an intense ~500 bp and two (~750 bp and ~450 bp) lighter U3 bands was shared by B-cells derived from all lymphoid organs examined. In contrast, four distinct U3 bands, ranging from ~500 bp to ~750 bp, were amplified in T-cells with variable intensity depending on the source lymphoid organs. It was interesting to note that the ~500 bp U3 band was more intense in B-cells from all four LN samples compared to B-cells from the spleen and thymus. The findings from this study suggest that the expression of MuLV-ERVs is differentially regulated depending on the lymphocyte type, source lymphoid organ, and ERV type.

### Burn-elicited alterations in MuLV-ERV expression in lymphocytes depending on lymphocyte type, source lymphoid organ, and ERV type

**Figure 2:**
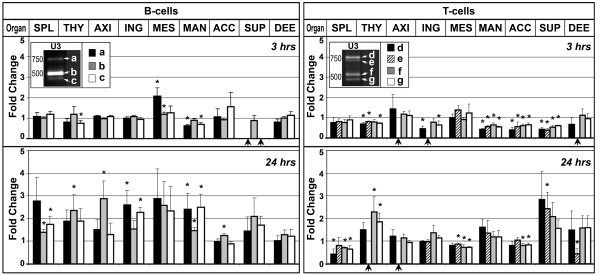
**Post-burn alterations in the expression of MuLV-ERVs in B- and T-cells isolated from lymphoid organs of C57BL/6J mice.** Post-burn (3 hours and 24 hours) changes in the expression of MuLV-ERVs were measured in the B- and T-cells isolated from nine different lymphoid organs. The results, which were presented as a fold change (burn/no burn) for the individual MuLV-ERV U3 bands (a, b, and c for B-cells; d, e, f, and g for T-cells), revealed a unique pattern for each experimental group (cell/organ/time). SPL (spleen), THY (thymus), LN (lymph node), AXI (axillary LN), ING (inguinal LN), MES (mesenteric LN), MAN (mandibular cervical LN), ACC (accessory mandibular cervical LN), SUP (superficial parotid cervical LN), DEE (deep cervical LN). A black arrow indicates unmeasured samples due to the low expression level. Error bars indicate standard error of the mean. * P < 0.05.

The effects of burn-elicited stressors on the expression of MuLV-ERVs in the B- and T-cells from the spleen, thymus, and seven lymph nodes were examined at 3 hours and 24 hours after injury by amplifying the 3^′^ U3 regions. At 3 hours post-burn, the MuLV-ERV expression, represented by four distinct U3 bands, was decreased in the T-cells from the thymus and three cervical LNs (mandibular, accessory mandibular, and superficial parotid), but not in the T-cells from the deep cervical LN (Figure 2). In contrast, there was an increase in intensity of one to two U3 bands in the T-cells from the thymus and superficial parotid cervical LN at 24 hours post-burn when there was a decrease in U3 bands in the T-cells of the spleen and mesenteric LN.

In B-cells, there was an induction of two (a and b bands) of the three U3 bands in the mesenteric LN at 3 hours post-burn while two bands (a and c) were down regulated in the B-cells of the mandibular cervical LN. However, at 24 hours post-burn, the expression levels of all three U3 bands were increased in the B-cells from the mandibular cervical LN. Also at the 24 hours time point, post-burn increases in one or two U3 bands were also observed in the B-cells of the spleen, thymus, axillary LN, inguinal LN, and/or accessory mandibular cervical LN.

The results from this study suggest that the lymphocyte type (B- or T-cell), source lymphoid organ, and duration after injury play a role in post-burn changes (induction or repression) in the expression of MuLV-ERVs. It is possible that the post-burn differential expression of MuLV-ERVs resulted from changes in the composition of the B- and T-cell sub-populations (*e.g.* naïve vs. effector cells), which have different transcription environments, instead of (and/or in addition to) the affected MuLV-ERVs’ altered transcriptional potentials. It was interesting to note that the post-burn increase in the MuLV-ERV expression in the B-cells from the mesenteric LN parallels the proliferation of specific B-cell populations in the mesenteric LN and spleen after burn [23].

### MuLV-ERV U3 sequences identified in specific lymphocyte type and/or injury (no burn/burn) condition

**Figure 3:**
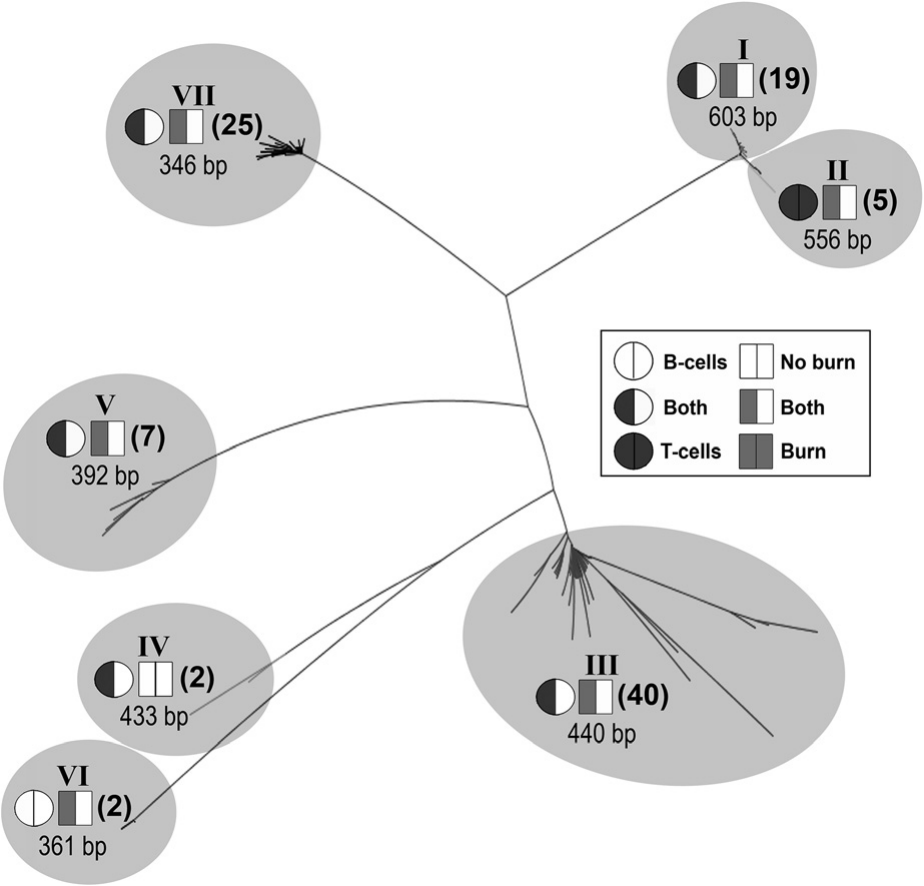
**Phylogenetic relatedness of 100 unique MuLV-ERV U3 sequences.** Phylogenetic relatedness of the 100 unique MuLV-ERV U3 sequences was determined by a multiple alignment analysis. The MuLV-ERV U3 sequences in each branch were analyzed for their presence in a specific lymphocyte type (B- or T-cell) and/or stress condition (no burn or burn). Seven branches were formed, primarily based on the size of the U3 sequences. Number of sequences is in parentheses.

Three clones from each of the 88 MuLV-ERV U3 amplicons/bands, which showed contrasting expression levels between the individual no burn and burn pairs, were sequenced to identify lymphocyte type- and/or injury (no burn/burn)-specific U3 sequences. From a total of 260 MuLV-ERV U3 sequences cloned from the entire set of MuLV-ERV U3 bands examined, 100 unique U3 sequences, ranging from 346 nucleotides to 615 nucleotides in size, were identified (Additional file 1: Table S1). An initial phylogenetic analysis resulted in seven distinct branches of U3 sequences, primarily segregated by their size (group I [largest U3s] ~ group VII [smallest U3s]) (Figure 3). Group III (440 bp length) had the most number of U3 sequences (40) followed by group VII (346 bp length) (25 sequences) then group I (greater than 603 bp length) (19 sequences). There were only two U3 sequences in groups IV and VI (433 bp and 361 bp in size, respectively). The group II U3 sequences (5) were isolated only from T-cells and the group VI U3 sequences (2) were unique for B-cells. On the other hand, the group IV U3 sequences (2) were identified only from no burn control mice. The other four branches (groups I, III, V, and VII) consisted of U3 sequences isolated from both lymphocyte types derived from both no burn and burn mice.

The results obtained from this study suggest that some MuLV-ERVs are selectively expressed depending on lymphocyte type (B- or T-cell) and/or presence of burn-elicited stressors. However, it is possible that an expanded saturation cloning protocol would find that some cell type and/or burn-specific U3 sequences are not exclusively present in one group over the other. In this case, a statistical evaluation may be needed to determine the specificity of the U3 sequences in regard to the lymphocyte type and/or burn-elicited stressors.

### Characteristics of seven U3 sequences representing the phylogenetic branches

Seven representative U3 sequences, which were chosen from the seven main phylogenetic branches, were subjected to an alignment analysis using reference sequences to characterize their sequence features, such as direct repeats, unique sequence, and an insertion of 190 nucleotides. These U3 sequence features were utilized as key parameters for the *in silico* determination of the MuLV-ERV tropism traits [19]. The U3 sequences of the groups I and II had an insertion of 190 nucleotides, which is consistent with their relatively large size compared to the other five branches (Table 1). There was a well-conserved TATA box in all seven U3 sequences; however, each U3 sequence retained a unique profile in regard to the direct repeats (1/1*, 4/4*, 5/5*, and 6/6*) and the unique sequence (2). The specific features of the individual U3 sequences were translated into the tropism traits of the respective putative MuLV-ERVs [17,19]. However, the sequences features of the group VI U3 sequences were too diverse to predict its tropism trait.

**Table 1:** Sequence characteristics and putative tropism traits of seven MuLV-ERV U3 sequences representing the seven main phylogenetic branches

**Group**	**U3 Probe**	**Size (bp)**	**Repeat/unique region**					**TATA**	**Insertion (190 bp)**	**Tropism**
			1/1*	**2**	4/4*	5/5*	6/6*			
I	B_3H_-T_TH_-1	603	+/+	+	+	+	+/+	+	+	P-II
III	B_3H_-T_IN_-3	556	+/+	·	+	+	+/+	+	+	P-I
III	B_3H_-T_TH_-7	440	+/+	+	+	+/+	+/+	+	·	X-II
IV	NB_24H_-T_SC_-3	433	+/+	·	+/+	+/+	+	+	·	X-II
V	B_3H_-T_TH_-6	392	+/+	+	+	+/+	+/+	+	·	X-I
VI	B_3H_-B_ME_-9	361	+/+	·	·	+/+	+/+	+	·	·
VII	B_3H_-T_TH_-11	346	+/+	+	+/+	·	+/+	+	·	X-II

Sequence characteristics: direct repeats (1/1*, 4/4*, 5/5*, and 6/6*), unique region (2), insertion of 190 nucleotides, and TATA box; +/+ (direct repeat), + (single copy), · (absence).

### Evidence for post-burn increase in the expression of specific MuLV-ERVs

**Figure 4:**
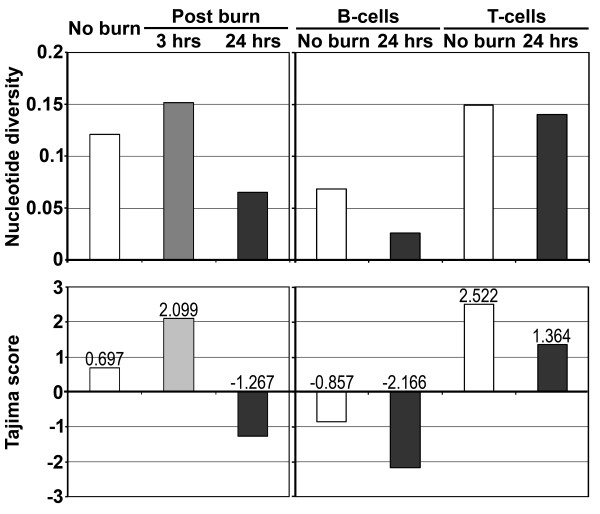
**Decreased population diversity of MuLV-ERV U3 sequences at 24 hours after burn.** The U3 population diversity within each experimental group was measured [21] and the relevant values, indicating different diversity levels, were presented as nucleotide diversity and Tajima statistics scores. There was a marked decrease in the U3 population diversity at 24 hours after burn compared to no burn.

The population diversity of the U3 sequences (a total of 260) was measured in each experimental group and compared among the groups in two different analyses [21]: i) no burn, burn-3 hours, and burn-24 hours, and ii) no burn-B-cells, burn-24 hours-B-cells, no burn-T-cells, and burn-24 hours-T-cells. The results from the first diversity analysis revealed that the U3 population of the burn-24 hours group was substantially less heterogeneous compared to the other two groups (no burn and burn-3 hours) (Figure 4). In contrast, it appears that there was an increase in diversity among the U3 population in the burn-3 hours group. The finding of decreased U3 population diversity within the burn-24 hours group was confirmed by its negative score for the Tajima’s Neutrality test in comparison to the positive values of the other two groups [24]. The results from the second diversity analysis demonstrated that there is a decrease in U3 population diversity in both burn-24 hours-B-cells and burn-24 hours-T-cells groups in comparison to the no burn controls. Furthermore, it was apparent that at the 24 hours time point, the U3 population in B-cells from both no burn and burn were less diverse in comparison to T-cells’ U3 population.

The findings from these diversity analyses indicate that burn-elicited stressors lead to the induction of specific MuLV-ERVs in a time after injury-dependent manner, and the population of MuLV-ERVs expressed in T-cells is more heterogeneous than the population in B-cells.

### Differential transcription potentials of MuLV-ERV U3-promoter sequences

The U3 sequence on the 5^′^ LTR, which is presumed to be identical to the U3 of the 3^′^ LTR, serves as an ERV promoter. In this study, the transcription potentials of the 100 unique U3-promoter sequences were surveyed for transcription regulatory elements (TREs). Nine TREs were predominantly present in the U3 sequences isolated from both B- and T-cells of burn mice at the 3 hours and/or 24 hours time points compared to the U3 sequences from no burn mice (Table 2 and Additional file 2: Table S2). Among the nine TREs are BRNF (Brn POU domain factors), AIRE (Autoimmune regulatory element binding factor), and NRF1 (Nuclear respiratory factor 1), which have been reported to be linked to various stress responses [25,26]. Furthermore, it appears that three TREs occurred more often in the U3 sequences of B-cells than T-cells whereas the U3 sequences from T-cells had a higher frequency of 16 other TREs in comparison to B-cells (Table 2 and Additional file 2: Table S2). Among the 16 TREs, MAZF (Myc associated zinc fingers), GATA (GATA binding factors) and MEF2 (myocyte-specific enhancer binding factor) have been reported to be involved in T-cell activation [27-29]. A total of 122 TREs, including the 28 TREs described above, were identified from the 100 unique sequences, and the comprehensive TRE profiles were summarized in Additional file 2: Table S2 and Additional file 3: Table S3.

**Table 2:** Differential transcription potentials of MuLV-ERV U3-promoter sequences

**TRE**	**Consensus motif**	**Occurrence**		
		**Burn/No burn**		
**DMRT**	ctttctt**GTAA**cttttgagaa	68	/	1
**BRNF**	tctggatctat**TGAT**ttga	35	/	4
**BCL6**	ctctttc**TGGA**aactga	31	/	3
**TF2D**	gctctggtactttttcatcccttgcaaaat**GGCG**ttact	27	/	1
**PTF1**	gcca**TCTG**ttcttggccctga	28	/	1
**MYT1**	tgag**AGCT**cagct	27	/	2
**AIRE**	gtatttctcggtcattt**GGGG**aaactg	19	/	0
**ZNFP**	caca**CACC**tggtc	22	/	0
**NRF1**	ggg**GCCC**aggcgcttga	21	/	1
**TRE**	**Consensus motif**	**Occurrence**		
		**B-cell/T-cell**		
**PAX2**	gttattcggggaacctg**AAAC**tg	32	/	13
**HAML**	ctga**GTGG**ttagttc	35	/	8
**NKXH**	acttttga**GAAC**ttagctc	33	/	10
**HOMF**	taatct**TTAA**ctgttaatc	16	/	37
**SORY**	gttctaaa**CAAT**gtctattttca	14	/	28
**HEAT**	gagagggtctaat**GAAG**gttcaaca	10	/	30
**MAZF**	atgg**GAGG**ggtac/gtac**GAGG**agagg	8	/	18
**MEF2**	acaatgt**CTAT**tttcaagaaatg	5	/	19
**GATA**	ttt**AGAT**ctaaac	6	/	16
**CART**	gggtc**TAAT**gaaggttcaaca	8	/	16
**HOXF**	gggtc**TAAT**gaaggttcaa	7	/	17
**LHXF**	gagagggtc**TAAT**gaaggttcaa	7	/	16
**FXRE**	**AGGT**gcttgacca	5	/	19
**GRHL**	cacaca**GGTT**caa	7	/	16
**NKX6**	atct**TTAA**ctgttaa	6	/	16
**CEBP**	aaggttca**GCAA**tgg	6	/	15
**FKHD**	tagttc**TAAA**caatgtc	5	/	16
**PDX1**	gaaagagtc**TAAT**gaaggt	5	/	15
**RXRF**	cacag**GTTC**aaggagtggccagagc	5	/	16

Numbers indicate the occurrence frequency of specific TREs (transcription regulatory elements) within the 100 unique U3-promoter sequences. Please refer to (Additional file 3: Table S3) for the complete list of TRE abbreviations.

It is probable that the TREs (*e.g.*, BRNF, AIRE, NRF1), which were mapped on the U3 sequences almost exclusively derived from burn mice, are closely associated with the modulation of MuLV-ERV expression in conjunction with a transcription environment dynamically formulated by burn-elicited stressors.

### *In silico* cloning and characterization of putative MuLV-ERVs harboring the unique U3 sequences

To investigate the biological properties of the putative MuLV-ERVs harboring the U3 sequences identified in this study, the C57BL/6J mouse genome was surveyed to map the proviral loci using the 100 unique U3 sequences as mining probes. From the *in silico* mining experiment, 49 ERVs were found with two identical LTR sequences at both ends and two, MuERV-49 and MuERV-26, retained slightly different LTR sequences due to a few point mutations. Forty-nine of the 51 putative MuLV-ERVs identified from this survey, including 15 full-length proviruses retaining a set of intact coding potentials for *gag, pol* and *env* polypeptides, have been reported in previous studies [14,30-33]. The two newly identified MuLV-ERVs are: one full-length provirus (8,759 nucleotides) with intact coding potentials and one defective provirus (6,846 nucleotides) (Table 3). An analysis of the primer binding site revealed that both MuLV-ERVs had the tRNA^Gln^ priming sequence. All 51 putative MuLV-ERVs were mapped using the U3 probes derived from both no burn and burn mice, except for BM-a-2.11e [31], which was identified using a U3 probe unique for B-cells of burn mice. On the other hand, 30 putative MuLV-ERV loci were retrieved using U3 probes exclusively isolated from T-cells. Future studies focusing on the biological properties of these putative MuLV-ERVs would be of interest: 1) effects (*e.g.,* cytotoxicity) of their replication on immune cells in conjunction with infection followed by random integration into the genome and 2) functions (*e.g.*, regulation of inflammatory mediators) of their gene products (*gag*, *pol*, and *env* polypeptides).

**Table 3:** Genomic map and characteristics of putative MuLV-ERVs harboring the unique U3 sequences

**No**	**Virus**	**Chr**	**Location**		**Ori**	**Virus size (bp)**	**PBS**	**ORF**			**Occurrence frequency**	**Reference**
			**From**	**To**				**gag**	**pol**	**env**		
1	MuERV-49	1	133539144	133548197	+	9054	Q	-	+	+	U/U	I,II,III,**IV**
2	MuERV-31	1	166153938	166159683	+	5746	Q	+	-	-	U/T	I,II,**IV**
3	MuERV-30	1	184187251	184196231	+	8981	Q	+	P	+	U/T	I,II,**IV**
4	MuERV-33	1	193757492	193766472	+	8981	Q	+	P	-	U/T	I,II,**IV**
5	MuERV-35	2	57066823	57075803	-	8981	Q	+	+	+	U/T	I,II,**IV**
6	MuERV-3	2	156182292	156189341	-	7050	Q	+	-	+	U/U	III,**IV**
7	MuERV-48	3	66900000	66907716	+	7717	Q	+	-	-	U/U	I,II,III,**IV**
8	MuERV-55	3	151986104	151995146	+	9043	Q	+	+	+	U/U	I,II,III,**IV**
9	MuERV-29	4	101535762	101544743	-	8982	Q	+	P	+	U/T	I,II,**IV**
10	MuERV-28	4	107818639	107827621	-	8983	Q	+	+	+	U/T	I,II,**IV**
11	MuERV-2	4	132651872	132657538	+	5667	Q	-	-	-	U/U	I,II,III,**IV**
12	MuERV-6	4	133715306	133720617	+	5312	Q	+	-	-	U/U	I,II,III,**IV**
13	MuLV_ERV_4-5_*	4	145686198	145694956	-	8759	Q	+	+	+	U/U	this study
14	XMV9	4	146592937	146601658	+	8722	Q	+	+	+	U/U	**V**
15	L-3-9.5	5	23206397	23215063	-	8667	P	+	+	+	U/U	I,**II**
16	MuLV_ERV_5-2_*	5	23721126	23727971	-	6846	Q	+	-	+	U/U	this study
17	MuERV-27	5	33864636	33873616	+	8981	Q	-	+	+	U/T	I,II,**IV**
18	MuERV-18	5	44525599	44534580	+	8982	Q	-	+	+	U/T	I,II,**IV**
19	MuERV-19	5	77359541	77368522	+	8982	Q	+	+	+	U/T	I,II,**IV**
20	MuERV-26	7	7022622	7031603	-	8982	Q	P	-	-	U/T	I,II,**IV**
21	MuERV-24	7	30400226	30408924	+	8699	Q	+	+	P	U/T	I,II,**IV**
22	MuERV-25	7	31473659	31482639	+	8981	Q	+	P	-	U/T	I,II,**IV**
23	MuERV-34	7	123575623	123581296	+	5674	Q	+	-	-	U/T	I,II,**IV**
24	MuERV-10	8	44397084	44405817	-	8734	Q	+	+	+	U/T	I,II,**IV**
25	MuERV-11	8	87649754	87656141	-	6388	Q	+	P	-	U/T	I,II,**IV**
26	MuERV-1	8	93410190	93415857	+	5668	Q	-	-	-	U/U	I,II,III,**IV**
27	MuERV-12	8	122413465	122420940	-	7476	Q	-	-	+	U/T	I,II,**IV**
28	MuERV-8	9	41713568	41720351	-	6784	Q	-	+	+	U/U	I,II,**IV**
29	iLN(III)-1a	9	62286981	62295739	+	8759	Q	+	+	+	U/U	**I**
30	K-4-11.10a	10	8263933	8271066	-	7134	Q	-	-	-	U/T	I **II**
31	MuERV-53	10	22454084	22463128	+	9045	Q	+	+	+	U/U	I,II,III,**IV**
32	MuERV-17	10	41156454	41165434	+	8981	Q	+	+	+	U/T	I,II,**IV**
33	BM-a-2.11e	11	5905655	5911518	+	5864	Q	-	-	-	B/B	**III**
34	MuERV-22	11	6649425	6658394	-	8970	Q	+	-	+	U/T	I,II,**IV**
35	MuERV-23	11	820301	8829280	+	8980	Q	+	+	+	U/T	I,II,**IV**
36	MuERV-50	11	60392151	60399510	-	7360	Q	-	P	-	U/U	I,II,III,**IV**
37	MuERV-51	11	76362310	76371354	+	9045	Q	+	+	+	U/U	I,II,III,**IV**
38	MuERV-38	11	88751494	88758280	-	6787	Q	+	-	+	U/T	I,II,**IV**
39	MuERV-37	12	20704753	20710513	+	5761	Q	-	-	-	U/T	I,II,**IV**
40	MuERV-16	12	55875211	55884192	+	8982	Q	+	+	+	U/T	**IV**
41	MuERV-5	13	67985825	67994511	-	8687	Q	+	+	+	U/U	I,II,III,**IV**
42	MuERV-21	13	99756230	99765210	-	8981	Q	+	P	+	U/T	I,II,**IV**
43	MuERV-7	14	54912048	54917591	-	5544	Q	+	-	-	U/U	**IV**
44	L-1-2.14	14	55149203	55157025	-	7823	Q	P	+	+	U/U	I,**II**
45	MuERV-15	15	76390247	76397432	-	7186	Q	-	-	+	U/T	I,II,**IV**
46	U-1-5.16	16	76135288	76144269	-	8982	Q	-	P	-	U/T	I,**II**
47	MuERV-14	16	93697702	93706682	-	8981	Q	-	+	+	U/T	I,**IV**
48	MuERV-20	18	82862943	82871822	+	8880	Q	-	+	+	U/T	I,II,**IV**
49	MuERV-32	19	38449512	38456573	+	7062	Q	-	P	-	U/T	I,II,**IV**
50	MuERV-9	19	61001210	61009936	-	8727	Q	+	+	+	U/U	I,II,**IV**
51	MuERV-13	X	15051174	15060154	+	8981	Q	+	P	+	U/T	I,II,**IV**

Chr (chromosome), Ori (orientation), PBS (primer binding site), Q (tRNA^glutamine^), P (tRNA^proline^), ORF (open reading frame), + (intact/full-length), - (defective), U (both B- and T- cells; both no burn and burn), B (B-cell), T (T-cell), references I [14], II [33], III [31], IV [30] and V [32]. *MuLV-ERV newly identified in this study. The names of MuLV-ERVs, which have been reported in previous studies, are adapted from the relevant reference in bold.

### Identification of genes neighboring the MuLV-ERV loci

The U3 promoter and the enhancer sequences embedded in MuLV-ERVs can affect the transcriptional activities of the neighboring genes via the promotion/repression of transcription, and alternative splicing and polyadenylation [34]. In this study, we identified the genes located within 100 Kb upstream and downstream of each MuLV-ERV integration site. Of a total of 131 genes identified, nine genes had MuLV-ERVs integrated into their introns (Additional file 4: Table S4). It will be interesting to examine how the expression patterns (depending on lymphocyte type or burn-elicited stress) of these proviruses are correlated with the expression of the relevant genes, for example, the relationship between BM-a-2.11e MuLV-ERV, which was identified using a U3 probe uniquely identified from the B-cells of burn mice, and Camk2b (calcium/calmodulin-dependent protein kinase II-beta) locus. The Camk2b expression is associated with T-cell activation and cytotoxic T-cell activity [35,36]. It is possible that the burn-elicited stressors may affect the promoter and/or enhancer activities of certain MuLV-ERVs paralleling modulation of the expression of adjacent genes in a specific lymphocyte type.

## Conclusion

It is expected that burn injury elicits a range of stress signals that are directly linked to pathologic changes in the immune system and other organs [37]. The majority of the underlying mechanisms regarding how these stressors contribute to post-burn pathogenesis still remain to be characterized. The findings from this study suggest that certain MuLV-ERVs are differentially modulated depending on the lymphocyte type, location of lymphoid organ, and duration after injury in conjunction with the burn-elicited stressors. As a result of activation of certain MuLV-ERVs following burn injury, the gene products and/or newly produced virus particles of the activated ERVs may participate in specific burn pathogeneses, such as immune disorder and cytotoxicity, in a cell type-specific manner. In fact, our laboratory has reported that some MuLV-ERVs are activated in the liver of mice after burn injury which resulted in evident hepatic damage [33]. Further investigations are needed to elucidate the link between lymphocyte type-specific post-burn MuLV-ERV activation and systemic inflammatory disorder as well as the other aspects of burn pathogeneses.

## Abbreviations

HERV: Human endogenous retroviruses; LN: Lymph node; LTR: Long terminal repeat; MuLV-ERVs: Murine leukemia virus-type endogenous retroviruses; PBS: Primer binding site; TREs: Transcription regulatory elements.

## Competing interests

There is no conflicting financial interest.

## Authors’ contributions

This study was conceived and managed by KC. DGG participated in scientific discussions. KHL, DL, and TG performed the experiments. KHL generated the figures and drafted the manuscript. All authors read and approved the final manuscript.

## Supplementary Material

Additional file 1: Table S1 Sources of the 100 MuLV-ERV U3 sequences analyzed in this study. Yellow and gray highlights indicate unique MuLV-ERV U3 sequences and clones without sequence information, respectively. MAN (mandibular cervical LN), ACC (accessory mandibular cervical LN), SUP (superficial parotid cervical LN), DEE (deep cervical LN) LN (lymph node), SM (size marker). Click here for file

Additional file 2: Table S2 The profile of transcription regulatory elements in 100 unique MuLV-ERV U3-promoter sequences. The number in the box indicates the occurrence frequency of each transcription regulatory element (TRE). Colored boxes indicate TREs predominantly identified in the U3 sequences derived from specific experimental groups: burn group (green), B-cell group (yellow), and T-cell group (blue). Click here for file

Additional file 3: Table S3 Abbreviations: transcription regulatory elements. Click here for file

Additional file 4: Table S4 Annotated genes neighboring 100 Kb upstream and downstream of the individual MuLV-ERV integration loci. Chr (Chromosome number), Ori (Orientation). Gray highlight indicates the annotated genes harboring an ERV in the intron. *MuLV-ERV newly identified in this study. Please refer to Table 3 for the MuLV-ERV names. Click here for file
