# Supplementary material for: Injury-elicited stressors alter endogenous retrovirus expression in lymphocytes depending on cell type and source lymphoid organ
Source: BMC Immunol. 2013 Jan 5;14:2. doi: 10.1186/1471-2172-14-2 (PMC3562510; doi:10.1186/1471-2172-14-2)
Supplement: Additional file 1: Table S1 — Sources of the 100 MuLV-ERV U3 sequences analyzed in this study. Yellow and gray highlights indicate unique MuLV-ERV U3 sequences and clones without sequence information, respectively. MAN (mandibular cervical LN), ACC (accessory mandibular cervical LN), SUP (superficial parotid cervical LN), DEE (deep cervical LN) LN (lymph node), SM (size marker). [file 1471-2172-14-2-S1.pdf]

Table S1. Sources of the 100 MuLV-ERV U3 sequences analyzed in this study

Burn

Cell	Amplicon			Clone	Cell	Amplicon			Clone
B-cell	1	Time	3 hr	B1-1	T-cell	23	Time	3 hr	B23-1
		Tissue	THY	B1-2			Tissue	MAN	B23-4
		Band	c	B1-7			Band	f	B23-5
	2	Time	3 hr	B2-1		24	Time	3 hr	B24-2
		Tissue	MES	B2-2			Tissue	MAN	B24-3
		Band	a	B2-3			Band	g	B24-4
	3	Time	3 hr	B3-1		25	Time	3 hr	B25-1
		Tissue	MES	B3-3			Tissue	ACC	B25-2
		Band	b	B3-9			Band	d	B25-3
	4	Time	3 hr	B4-1		26	Time	3 hr	B26-1
		Tissue	MAN	B4-3			Tissue	ACC	B26-2
		Band	a	B4-4			Band	e	B26-3
	5	Time	3 hr	B5-1		27	Time	3 hr	B27-1
		Tissue	MAN	B5-2			Tissue	ACC	B27-2
		Band	c	B5-3			Band	f	B27-3
	6	Time	24 hr	B6-1		28	Time	3 hr	B28-1
		Tissue	SPL	B6-2			Tissue	ACC	B28-2
		Band	b	B6-3			Band	g	B28-3
	7	Time	24 hr	B7-1		29	Time	3 hr	B29-1
		Tissue	SPL	B7-2			Tissue	SUP	B29-2
		Band	c	B7-3			Band	d	B29-3
	8	Time	24 hr	B8-1		30	Time	3 hr	B30-1
		Tissue	THY	B8-2			Tissue	SUP	B30-2
		Band	b	B8-3			Band	e	B30-3
	9	Time	24 hr	B9-3		31	Time	3 hr	B31-1
		Tissue	AXI	B9-4			Tissue	SUP	B31-2
		Band	b	B9-5			Band	f	B31-3
	10	Time	24 hr	B10-1		32	Time	3 hr	B32-1
		Tissue	ING	B10-2			Tissue	SUP	B32-2
		Band	a	B10-3			Band	g	B32-3
	11	Time	24 hr	B11-1		33	Time	24 hr	B33-3
		Tissue	ING	B11-2			Tissue	SPL	B33-4
		Band	c	B11-3			Band	d	B33-5
	12	Time	24 hr	B12-1		34	Time	24 hr	B34-3
		Tissue	MAN	B12-2			Tissue	SPL	B34-4
		Band	a	B12-3			Band	f	B34-5
	13	Time	24 hr	B13-1		35	Time	24 hr	B35-1
		Tissue	MAN	B13-2			Tissue	SPL	B35-2
		Band	b	B13-3			Band	g	B35-3
	14	Time	24 hr	B14-4		36	Time	24 hr	B36-1
		Tissue	MAN	B14-5			Tissue	THY	B36-2
		Band	c	B14-6			Band	f	B36-5
	15	Time	24 hr	B15-3		37	Time	24 hr	B37-2
		Tissue	ACC	B15-4			Tissue	THY	B37-3
		Band	b	B15-5			Band	g	B37-4
T-cell	16	Time	3 hr	B16-2		38	Time	24 hr	B38-1
		Tissue	THY	B16-10			Tissue	MES	B38-2
		Band	d	B16-11			Band	e	B38-3
	17	Time	3 hr	B17-1		39	Time	24 hr	B39-1
		Tissue	THY	B17-4			Tissue	MES	B39-2
		Band	e	B17-5			Band	f	B39-3
	18	Time	3 hr	B18-3		40	Time	24 hr	B40-1
		Tissue	THY	B18-6			Tissue	THY	B40-2
		Band	g	B18-7			Band	g	B40-3
	19	Time	3 hr	B19-1		41	Time	24 hr	B41-1
		Tissue	ING	B19-2			Tissue	ACC	B41-2
		Band	d	B19-3			Band	f	B41-3
	20	Time	3 hr	B20-1		42	Time	24 hr	B42-1
		Tissue	ING	B20-4			Tissue	ACC	B42-2
		Band	g	B20-7			Band	g	B42-3
	21	Time	3 hr	B21-1		43	Time	24 hr	B43-4
		Tissue	MAN	B21-2			Tissue	SUP	B43-5
		Band	d	B21-3			Band	e	B43-6
	22	Time	3 hr	B22-1		44	Time	24 hr	B44-1
		Tissue	MAN	B22-2			Tissue	DEE	B44-2
		Band	e	B22-5			Band	e	B44-3

No burn

Cell	Amplicon			Clone	Cell	Amplicon			Clone
B-cell	45	Time	3 hr	N1-1	T-cell	67	Time	3 hr	N23-1
		Tissue	THY	N1-2			Tissue	MAN	N23-2
		Band	c	N1-4			Band	f	N23-3
	46	Time	3 hr	N2-1		68	Time	3 hr	N24-1
		Tissue	MES	N2-2			Tissue	MAN	N24-2
		Band	a	N2-3			Band	g	N24-3
	47	Time	3 hr	N3-1		69	Time	3 hr	N25-1
		Tissue	MES	N3-2			Tissue	ACC	N25-2
		Band	b	N3-3			Band	d	N25-3
	48	Time	3 hr	N4-2		70	Time	3 hr	N26-1
		Tissue	MAN	N4-4			Tissue	ACC	N26-2
		Band	a	N4-5			Band	e	N26-3
	49	Time	3 hr	N5-1		71	Time	3 hr	N27-1
		Tissue	MAN	N5-2			Tissue	ACC	N27-2
		Band	c	N5-3			Band	f	N27-3
	50	Time	24 hr	N6-1		72	Time	3 hr	N28-1
		Tissue	SPL	N6-2			Tissue	ACC	N28-2
		Band	b	N6-3			Band	g	N28-3
	51	Time	24 hr	N7-1		73	Time	3 hr	N29-1
		Tissue	SPL	N7-2			Tissue	SUP	N29-2
		Band	c	N7-3			Band	d	N29-3
	52	Time	24 hr	N8-1		74	Time	3 hr	N30-1
		Tissue	THY	N8-2			Tissue	SUP	N30-2
		Band	b	N8-3			Band	e	N30-3
	53	Time	24 hr	N9-1		75	Time	3 hr	N31-1
		Tissue	AXI	N9-2			Tissue	SUP	N31-2
		Band	b	N9-3			Band	f	N31-3
	54	Time	24 hr	N10-1		76	Time	3 hr	N32-1
		Tissue	ING	N10-2			Tissue	SUP	N32-2
		Band	a	N10-3			Band	g	N32-3
	55	Time	24 hr	N11-1		77	Time	24 hr	N33-1
		Tissue	ING	N11-2			Tissue	SPL	N33-2
		Band	c	N11-3			Band	d	N33-3
	56	Time	24 hr	N12-1		78	Time	24 hr	N34-1
		Tissue	MAN	N12-2			Tissue	SPL	N34-2
		Band	a	N12-3			Band	f	N34-3
	57	Time	24 hr	N13-1		79	Time	24 hr	N35-1
		Tissue	MAN	N13-2			Tissue	SPL	N35-2
		Band	b	N13-3			Band	g	N35-3
	58	Time	24 hr	N14-1		80	Time	24 hr	N36-1
		Tissue	MAN	N14-2			Tissue	THY	N36-2
		Band	c	N14-3			Band	f	N36-3
	59	Time	24 hr	N15-1		81	Time	24 hr	N37-1
		Tissue	ACC	N15-2			Tissue	THY	N37-2
		Band	b	N15-3			Band	g	N37-3
T-cell	60	Time	3 hr	N16-1		82	Time	24 hr	N38-1
		Tissue	THY	N16-2			Tissue	MES	N38-2
		Band	d	N16-3			Band	e	N38-3
	61	Time	3 hr	N17-1		83	Time	24 hr	N39-1
		Tissue	THY	N17-2			Tissue	MES	N39-2
		Band	e	N17-3			Band	f	N39-3
	62	Time	3 hr	N18-1		84	Time	24 hr	N40-1
		Tissue	THY	N18-2			Tissue	THY	N40-2
		Band	g	N18-3			Band	g	N40-3
	63	Time	3 hr	N19-1		85	Time	24 hr	N41-1
		Tissue	ING	N19-2			Tissue	ACC	N41-2
		Band	d	N19-3			Band	f	N41-3
	64	Time	3 hr	N20-1		86	Time	24 hr	N42-1
		Tissue	ING	N20-2			Tissue	ACC	N42-2
		Band	g	N20-3			Band	g	N42-3
	65	Time	3 hr	N21-1		87	Time	24 hr	N43-1
		Tissue	MAN	N21-2			Tissue	SUP	N43-2
		Band	d	N21-3			Band	e	N43-3
	66	Time	3 hr	N22-1		88	Time	24 hr	N44-1
		Tissue	MAN	N22-2			Tissue	DEE	N44-2
		Band	e	N22-3			Band	e	N44-3
