# Supplementary material for: Injury-elicited stressors alter endogenous retrovirus expression in lymphocytes depending on cell type and source lymphoid organ
Source: BMC Immunol. 2013 Jan 5;14:2. doi: 10.1186/1471-2172-14-2 (PMC3562510; doi:10.1186/1471-2172-14-2)
Supplement: Additional file 2: Table S2 — The profile of transcription regulatory elements in 100 unique MuLV-ERV U3-promoter sequences. The number in the box indicates the occurrence frequency of each transcription regulatory element (TRE). Colored boxes indicate TREs predominantly identified in the U3 sequences derived from specific experimental groups: burn group (green), B-cell group (yellow), and T-cell group (blue). [file 1471-2172-14-2-S2.pdf]

Table S2. The profile of transcription regulatory elements in 100 unique MuLV-ERV U3-promoter sequences

U3 source: Cell	U3 source: Injury	Unique U3s	STAT	RUSH	KLFS
Both	Both	T3-B22-5	3	2	3
		T3-B21-1	3	2	3
		T3-B16-2	3	3	2
		T3-B23-1	1	1	1
		T24-B34-5	4	2	
		T3-B19-1	1	1	2
	Burn	B3-B4-1	3	2	4
		T3-B20-4	1	1	2
B cell	Both	T3-B30-2	3	2	2
		T24-B38-1	3	3	2
	Burn	T24-B43-5	2	1	2
		B3-B1-2	2	2	2
		B24-B7-3	3	3	2
		B3-B2-3	3	3	2
		T3-B32-1	3	2	2
		T24-B43-4	3	2	2
		T24-B37-3	2	3	2
		B3-B2-1	3	3	2
		B3-B1-1	3	3	2
		B24-B6-2	3	3	2
		T24-B44-1	3	3	
		T24-B40-1	3	3	2
		B3-B1-7	3	2	2
		T3-B32-3	3	3	1
		T24-B38-3	2	3	2
		B3-B3-3	3	3	2
		B3-B3-1	1	1	2
		B3-B2-2	1	1	2
		B3-B5-2	1	1	2
		B3-B5-1	1	1	2
		B24-B8-2	3	3	2
		B3-B5-3	1	1	2
		T24-B42-1	1	1	2
		T24-B39-3	3	3	2
		T24-B39-2	3	3	2
		B24-B9-4	3	3	2
		B3-B3-9	3	3	2
		T24-B42-2	1	1	
		T24-B44-3	3	3	2
		B3-B4-4	1	1	2
		B3-B4-3	3	2	3
	No burn	B3-N2-2	2	2	3
		B3-N3-3	3	2	3
		B3-N2-1	3	2	3
		B3-N4-2	3	2	3
		B24-N6-2	3	3	2
		B24-N6-1	1	1	2
		B24-N10-2	3	3	1
		B24-N10-1	4	3	2
		B24-N15-3	3	2	2
		B24-N14-1	3	3	2
		B24-N13-1	3	3	2
		B24-N13-2	2	3	2
		B24-N15-1	3	3	2
		B24-N9-2	3	3	2
		B24-N9-1	3	1	2
		B24-N7-1	2	3	2
		B24-N10-3	2	3	2
		B3-N3-2	1	1	2
		B24-N12-3	1	1	2
T cell	Both	T3-B24-3	3	2	4
		T3-B25-2	3	2	4
		T3-B20-7	4	2	
	Burn	T3-B30-1	4	2	3
		T24-B33-4	3	3	2
		T24-B35-2	3	3	2
		T3-B22-1	4	2	
		T24-B35-1	4	2	
		T24-B36-1	1		3
		T3-B23-5	1	1	2
		T3-B23-4	1	1	2
		T3-B17-5	3	1	3
		T3-B18-3	3	2	1
		T24-B35-3	2	2	3
		T24-B36-5	4	2	3
		T3-B17-4	3	2	
		T3-B17-1	3	3	2
	No burn	T24-N35-3	2	2	3
		T3-N18-3	4	2	3
		T3-N16-2	2	2	3
		B3-N3-1	3	2	3
		T3-N19-3	3	2	3
		T3-N22-3	3	2	3
		T3-N20-1	3	2	3
		B24-N13-3	3	2	3
		B3-N4-2	3	2	3
		T24-N36-3	2	3	2
		B3-N4-5	3	2	4
		T3-N21-2	3	2	1
		B24-N15-2	3	3	1
		B24-N11-2	3	3	2
		B24-N7-2	3	3	2
		B24-N8-2	4	2	
		B24-N8-1	2	3	2
		B3-N1-4	1	1	2
		T24-N34-2	1	1	3
		T24-N33-3	1	1	2
		T24-N37-3	3	1	2
		T24-N38-1	1	1	2
		T24-N37-1	1	1	2

S1. Tl

Unique U3s	HOXH
T3-B22-5	
T3-B21-1	
T3-B16-2	
T3-B23-1	
T24-B34-5	
T3-B19-1	
B3-B4-1	1
T3-B20-4	
T3-B30-2	
T24-B38-1	
T24-B43-5	
B3-B1-2	
B24-B7-3	
B3-B2-3	
T3-B32-1	
T24-B43-4	
T24-B37-3	
B3-B2-1	
B3-B1-1	
B24-B6-2	
T24-B44-1	
T24-B40-1	
B3-B1-7	
T3-B32-3	
T24-B38-3	
B3-B3-3	
B3-B3-1	
B3-B2-2	
B3-B5-2	
B3-B5-1	
B24-B8-2	
B3-B5-3	
T24-B42-1	
T24-B39-3	
T24-B39-2	
B24-B9-4	
B3-B3-9	
T24-B42-2	
T24-B44-3	
B3-B4-4	
B3-B4-3	
B3-N2-2	
B3-N3-3	
B3-N2-1	
B3-N4-2	
B24-N6-2	
B24-N6-1	
B24-N10-2	
B24-N10-1	
B24-N15-3	
B24-N14-1	
B24-N13-1	
B24-N13-2	
B24-N15-1	
B24-N9-2	
B24-N9-1	
B24-N7-1	
B24-N10-3	
B3-N3-2	
B24-N12-3	
T3-B24-3	
T3-B25-2	
T3-B20-7	
T3-B30-1	
T24-B33-4	
T24-B35-2	
T3-B22-1	
T24-B35-1	
T24-B36-1	
T3-B23-5	
T3-B23-4	
T3-B17-5	
T3-B18-3	
T24-B35-3	
T24-B36-5	
T3-B17-4	
T3-B17-1	
T24-N35-3	
T3-N18-3	
T3-N16-2	
B3-N3-1	
T3-N19-3	
T3-N22-3	
T3-N20-1	
B24-N13-3	
B3-N4-2	
T24-N36-3	
B3-N4-5	
T3-N21-2	
B24-N15-2	
B24-N11-2	
B24-N7-2	
B24-N8-2	
B24-N8-1	
B3-N1-4	
T24-N34-2	
T24-N33-3	
T24-N37-3	
T24-N38-1	
T24-N37-1	
