# Supplementary material for: Injury-elicited stressors alter endogenous retrovirus expression in lymphocytes depending on cell type and source lymphoid organ
Source: BMC Immunol. 2013 Jan 5;14:2. doi: 10.1186/1471-2172-14-2 (PMC3562510; doi:10.1186/1471-2172-14-2)
Supplement: Additional file 3: Table S3 — Abbreviations: transcription regulatory elements. [file 1471-2172-14-2-S3.pdf]

Table S3. Transcription regulatory element abbreviations

ABDB	Abdominal-B type homeodomain transcription factors
AHRR	AHR-arnt heterodimers and AHR-related factors
AIRE	Autoimmune regulatory element binding factor
AP1R	MAF and AP1 related factors
AP2F	Activator protein 2
AP4R	AP4 and related proteins
ARID	AT rich interactive domain factor
BCDF	Bicoid-like homeodomain transcription factors
BCL6	POZ domain zinc finger expressed in B-Cells
BRAC	Brachyury gene, mesoderm developmental factor
BRN5	Brn-5 POU domain factors
BRNF	Brn POU domain factors
CAAT	CCAAT binding factors
CART	Cart-1 (cartilage homeoprotein 1)
CDEF	Cell cycle regulators: Cell cycle dependent element
CDXF	Vertebrate caudal related homeodomain protein
CEBP	Ccaat/Enhancer Binding Protein
CIZF	CAS interating zinc finger protein
CLOX	CLOX and CLOX homology (CDP) factors
CP2F	CP2-erythrocyte Factor related to drosophila Elf1
CREB	cAMP-responsive element binding proteins
CSEN	Calsenilin, presenilin binding protein, EF hand transcription factor
CTCF	CTCF and BORIS gene family, transcriptional regulators with 11 highly conserved zinc finger domains
DEAF	Homolog to deformed epidermal autoregulatory factor-1 from D. melanogaster
DMRT	DM domain-containing transcription factors
E2FF	E2F-myc activator/cell cycle regulator
E4FF	Ubiquitous GLI - Krueppel like zinc finger involved in cell cycle regulation
EBOX	E-box binding factors
EGRF	EGR/nerve growth factor induced protein C & related factors
ETSF	Human and murine ETS1 factors
EVI1	EVI1-myleoid transforming protein
FAST	FAST-1 SMAD interacting proteins
FKHD	Fork head domain factors
FXRE	Farnesoid X - activated receptor response elements
GATA	GATA binding factors
GCMF	Chorion-specific transcription factors with a GCM DNA binding domain
GFI1	Growth factor independence transcriptional repressor
GLIF	GLI zinc finger family
GREF	Glucocorticoid responsive and related elements
GRHL	Grainyhead-like transcription factors
GTBX	GT box
GZF1	GDNF-inducible zinc finger gene 1
HAML	Human acute myelogenous leukemia factors
HAND	Twist subfamily of class B bHLH transcription factors
HBOX	Homeobox transcription factors
HEAT	Heat shock factors
HESF	Vertebrate homologues of enhancer of split complex
HIFF	Hypoxia inducible factor, bHLH/PAS protein family
HMTB	Human muscle-specific Mt binding site
HNF1	Hepatic Nuclear Factor 1
HNF6	Onecut homeodomain factor HNF6
HOMF	Homeodomain transcription factors
HOXC	HOX - PBX complexes
HOXF	Paralog hox genes 1-8 from the four hox clusters A, B, C, D
HOXH	HOX - MEIS1 heterodimers
HZIP	Homeodomain-leucine zipper transcription factors
IKRS	Ikaros zinc finger family
INRE	Core promoter initiator elements
INSM	Insulinoma associated factors
IRFF	Interferon regulatory factors
KLFS	Krueppel like transcription factors
LEFF	LEF1/TCF
LHXF	Lim homeodomain factors
MAZF	Myc associated zinc fingers
MEF2	MEF2, myocyte-specific enhancer binding factor
MOKF	Mouse Krueppel like factor
MYBL	Cellular and viral myb-like transcriptional regulators
MYOD	Myoblast determining factors
MYT1	MYT1 C2HC zinc finger protein
MZF1	Myeloid zinc finger 1 factors
NEUR	NeuroD, Beta2, HLH domain
NF1F	Nuclear factor 1
NFAT	Nuclear factor of activated T-cells
NFKB	Nuclear factor kappa B/c-rel
NKX6	NK6 homeobox transcription factors
NKXH	NKX homeodomain factors
NOLF	Neuron-specific-olfactory factor
NR2F	Nuclear receptor subfamily 2 factors
NRF1	Nuclear respiratory factor 1
NRSF	Neuron-restrictive silencer factor
OCT1	Octamer binding protein
OVOL	OVO homolog-like transcription factors
PARF	PAR/bZIP family
PAX2	PAX-2 binding sites
PAX3	PAX-3 binding sites
PAX6	PAX-4/PAX-6 paired domain binding sites
PAX8	PAX-2/5/8 binding sites
PBXC	PBX1 - MEIS1 complexes
PDX1	Pancreatic and intestinal homeodomain transcription factor
PIT1	GHF-1 pituitary specific pou domain transcription factor
PLAG	Pleomorphic adenoma gene
PRDF	Positive regulatory domain I binding factor
PREB	PREB core-binding element
PTF1	Pancreas transcription factor 1, heterotrimeric transcription factor
RBPF	RBPJ - kappa
RORA	v-ERB and RAR-related orphan receptor alpha
RP58	RP58 (ZFP238) zinc finger protein
RU49	Zinc finger transcription factor RU49, zinc finger proliferation 1 - Zipro1
RUSH	SWI/SNF related nucleophosphoproteins with a RING finger DNA binding motif
RXRF	RXR heterodimer binding sites
SIXF	Sine oculis (SIX) homeodomain factors
SNAP	snRNA-activating protein complex
SORY	SOX/SRY-sex/testis determinig and related HMG box factors
SP1F	GC-Box factors SP1/GC
SPZ1	Testis-specific bHLH-Zip transcription factors
SRFF	Serum response element binding factor
STAT	Signal transducer and activator of transcription
TALE	TALE homeodomain class recognizing TG motifs
TCFF	TCF11 transcription factor
TEAF	TEA/ATTS DNA binding domain factors
TF2B	RNA polymerase II transcription factor II B
TF2D	General transcription factor IID, GTF2D
VSMA	Vertebrate SMAD family of transcription factors
VTBP	Vertebrate TATA binding protein factor
XBBF	X-box binding factors
XCPE	Activator-, mediator- and TBP-dependent core promoter element for RNA polymerase II transcription from TATA-less promoters
YBXF	Y-box binding transcription factors, multifunctional proteins involved in transcriptional and translational regulation, mRNA splicing, DNA replication and repair
YY1F	Activator/repressor binding to transcription initiation site
ZBPF	Zinc binding protein factors
ZFHX	Two-handed zinc finger homeodomain transcription factors
ZFXY	Zfx and Zfy - transcription factors implicated in mammalian sex determination
ZNFP	Zinc finger proteins
