# Supplementary material for: Injury-elicited stressors alter endogenous retrovirus expression in lymphocytes depending on cell type and source lymphoid organ
Source: BMC Immunol. 2013 Jan 5;14:2. doi: 10.1186/1471-2172-14-2 (PMC3562510; doi:10.1186/1471-2172-14-2)
Supplement: Additional file 4: Table S4 — Annotated genes neighboring 100 Kb upstream and downstream of the individual MuLV-ERV integration loci. Chr (Chromosome number), Ori (Orientation). Gray highlight indicates the annotated genes harboring an ERV in the intron. *MuLV-ERV newly identified in this study. Please refer to Table 3 for the MuLV-ERV names. [file 1471-2172-14-2-S4.pdf]

**Table S4. Annotated genes neighboring 100 Kb upstream and downstream of the individual MuLV-ERV integration loci**

| Chr | Virus | Gene | Ori Virus/ Gene | Gene description |
|---|---|---|---|---|
| 1 | MuERV-49 | Avpr1b | (+/+) | arginine vasopressin receptor 1B |
| | | Slc26a9 | (+/+) | solute carrier family 26, member 9 |
| | | Ctse | (+/+) | cathepsin E |
| | MuERV-31 | Selp | (+/+) | selectin, platelet |
| | | F5 | (+/+) | coagulation factor V |
| | | Slc19a2 | (+/+) | solute carrier family 19 (thiamine transporter), member 2 |
| | | Blzf1 | (+/-) | basic leucine zipper nuclear factor 1 |
| | | Nme7 | (+/+) | non-metastatic cells 7 |
| | MuERV-33 | Slc30a1 | (+/+) | solute carrier family 30 (zinc transporter), member 1 |
| | | Traf5 | (+/+) | Tnf receptor-associated factor 5 |
| 2 | MuERV-35 | Gpd2 | (-/+) | nuclear receptor subfamily 4, group A, member 2 |
| | | Nr4a2 | (-/-) | glycerol phosphate dehydrogenase 2, mitochondrial |
| | MuERV-3 | Phf20 | (-/+) | PHD finger protein 20 |
| | | Scand1 | (-/+) | SCAN domain-containing 1 |
| | | Epb4.111 | (-/+) | erythrocyte protein band 4.1-like 1 |
| 3 | MuERV-48 | Rsrc1 | (+/+) | arginine/serine-rich coiled-coil 1 |
| | MuERV-55 | Nexn | (+/+) | nexilin |
| | | Usp33 | (+/+) | zinc finger, ZZ domain containing 3 |
| | | Zzz3 | (+/+) | ubiquitin specific peptidase 33 |
| 4 | MuERV-29 | Lepr | (-/+) | leptine receptor |
| | MuERV-2 | Fgr | (+/+) | gardner-rasheed feline sarcoma viral oncogene homolog |
| | | Ahdc1 | (+/+) | AT hook, DNA binding motif, containing 1 |
| | | Wasf2 | (+/+) | WAS protein family, member 2 |
| | MuERV-6 | Aim1l | (+/+) | absent in melanoma 1-like |
| | | Cd52 | (+/-) | CD52 antigen |
| | | Ubxd5 | (+/+) | UBX domain protein 11 |
| | | Sh3bgr13 | (+/-) | SH3 domain binding glutamic acid-rich protein-like 3 |
| | | Ccdc21 | (+/-) | coiled-coil domain containing 21 |
| | | Catsper4 | (+/-) | cation channel, sperm associated 4 |
| | | Cnksr1 | (+/-) | connector enhancer of kinase suppressor of Ras 1 |
| | | Zfp593 | (+/-) | zinc finger protein 593 |
| | | Grrp1 | (+/-) | glycine/arginine rich protein 1 |
| | MuERV-28 | Scp2 | (-/-) | sterol carrier protein 2, liver |
| | | Echdc2 | (-/+) | enoyl coenzyme A hydratase domain containing 2 |
| | | Zyg11a | (-/-) | zyg-11 homolog A (C. elegans) |
| | | Zyg11b | (-/-) | zyg-ll homolog B (C. elegans) |
| 5 | *MuLV-ERV$_{5-2}$ | Nupl2 | (-/+) | nucleoporin like 2 |
| | | Kcnh2 | (-/-) | potassium voltage-gated channel, subfamily H , member 2 |
| | | Klhl7 | (-/+) | kelch-like 7 |
| | MuERV-27 | Tmem130 | (+/-) | transmembrane protein 130 |
| | | Trrap | (+/-) | transformation/transcription domain-associated protein |
| | MuERV-18 | Prom1 | (+/-) | prominin 1 |
| | | Tapt1 | (+/-) | transmembrane anterior posterior transformation 1 |
| | MuERV-19 | Ppat | (+/-) | phosphoribosyl pyrophosphate amidotransferase |
| | | Paics | (+/+) | phosphoribosylaminoimidazole carboxylase |
| | | Srp72 | (+/+) | signal recognition particle 72 |
| | | Ar19 | (+/+) | ADP-ribosylation factor-like 9 |
| 7 | MuERV-24 | Wbp7 | (+/-) | WW domain binding protein 7 |
| | | Zbtb32 | (+/-) | zinc finger and BTB domain containing 32 |
| | | Upk1a | (+/-) | uroplakin 1A |
| | | Cox6b1 | (+/-) | cytochrome c oxidase, subunit VIb polypeptide 1 |
| | | Etv2 | (+/-) | ets variant gene 2 |
| | | Atp4a | (+/+) | ATPase, H+/K+ exchanging, gastric, alpha polypeptide |
| | | Tmem147 | (+/-) | transmembrane protein 147 |
| | | Gapdhs | (+/-) | glyceraldehyde-3-phosphate dehydrogenase |
| | | Sbsn | (+/+) | suprabasin |
| | | Dmkn | (+/+) | dermokine |
| | MuERV-25 | Krtdap | (+/+) | keratinocyte differentiation associated protein |
| | MuERV-34 | Pik3c2a | (+/-) | phosphatidylinositol 3-kinase, C2 domain, α polypeptide |
| | | Nucb2 | (+/+) | nucleobindin 2 |
| 8 | MuERV-10 | Zfp42 | (-/-) | zinc finger protein 42 |
| | MuERV-11 | Tnpo2 | (-/+) | transportin 2 (import 3, karyopherin beta 2b) |
| | | Fbxw9 | (-/+) | F-box and WD-40 domain protein 9 |
| | | Dhps | (-/+) | deoxyhypusine synthase |
| | | Man2b1 | (-/+) | mannosidase 2, alpha B1 |
| | MuERV-1 | Chd9 | (+/+) | chromodomain helicase DNA binding protein 9 |
| | MuERV-12 | Cotl1 | (-/-) | coactosin-like 1 (Dictyostelium) |

| Chr | Virus | Gene | Ori Virus/ Gene | Gene description |
|---|---|---|---|---|
| 8 | MuERV-12 | Usp10 | (-/+) | ubiquitin specific peptidase 10 |
| | | Crispld2 | (-/+) | cysteine-rich secretory protein LCCL domain containing 2 |
| 10 | K4-11.10a | Ust | (-/-) | uronyl-2-sulfotransferase |
| | MuERV-53 | Slc2a12 | (+/+) | solute carrier family 2, member 12 |
| | | Tbpl1 | (+/-) | TATA box binding protein-like 1 |
| | | Tcf21 | (+/-) | transcription factor 21 |
| | MuERV-17 | Zbtb24 | (+/+) | zinc finger and BTB domain containing 24 |
| | | Mical1 | (+/+) | microtubule associated monoxygenase |
| | | Smpd2 | (+/-) | sphingomyelin phosphodiesterase 2 |
| | | Ppill6 | (+/+) | peptidylprolyl isomerase (cyclophilin)-like 6 |
| | | Cd164 | (+/+) | CD164 antigen |
| 11 | MuERV-22 | Ramp3 | (-/+) | receptor (calcitonin) activity modifying protein 3 |
| | MuERV-23 | Pkd1/1 | (+/-) | polycystic kidney disease 1 like 1 |
| | | Hus1 | (+/-) | Hus1 homolog (S. pombe) |
| | | Sunc1 | (+/-) | Sad1 and UNC84 domain containing 1 |
| | MuERV-50 | NMyo15 | (-/+) | myosin XV |
| | | Alkbh5 | (-/+) | alkB, alkylation repair homolog 5 (E.coli) |
| | MuERV-51 | Abr | (+/-) | active BCR-related gene |
| | MuERV-38 | Akap1 | (-/-) | A kinase (PRKA) anchor protein 1 |
| | | Scpep1 | (-/-) | serine carboxypeptidase 1 |
| | | Coi1 | (-/+) | coilin |
| | BM-a-2.11e | Camk2b | (+/-) | calcium/calmodulin-dependent protein kinase II, beta |
| 12 | MuERV-16 | Eapp | (+/-) | E2F-associated phosphoprotein |
| | | Snx6 | (+/-) | sorting nexin 6 |
| | | Cf12 | (+/-) | cofilin 2, muscle |
| 13 | MuERV-5 | Zfp273 | (-/+) | zinc finger protein 273 |
| | MuERV-21 | Tnpo1 | (-/-) | transportin 1 |
| 14 | MuERV-7 | Mmp14 | (+/+) | matrix metallopeptidase 14 (membrane-insert |
| | | Lrp10 | (+/+) | low-density lipoprotein receptor-related protein 10 |
| | | Rem2 | (+/+) | rad and gem related GTP binding protein 2 |
| | | Prmt5 | (+/-) | protein arginine N-methyltransferase 5 |
| | | D14Ertd5 | (+/-) | DNA segment, Chr 14, ERATO Doi 500, expressed |
| | | Jub | (+/-) | ajuba |
| | | Psmb5 | (+/-) | proteasome subunit beta type 5 precursor |
| | | cdh24 | (+/-) | cadherin-like 24 |
| | L-1-2.14 | Dad1 | (-/-) | zinc finger protein 273 |
| | | Abhd4 | (-/+) | abhydrolase domain containing 4 |
| | | Olfr49 | (-/-) | olfactory receptor 49 |
| | | Oxa1l | (-/+) | oxidase assembly 1-like |
| | | Slc7a7 | (-/-) | solute carrier family 7 (cationic amino acid transporter,y+ system), member 7 |
| 15 | MuERV-15 | Bop1 | (-/-) | block of proliferation 1 |
| | | Msf1 | (-/+) | heat shock factor 1 |
| | | Dgat1 | (-/-) | diacylglycerol O-acyltransferase 1 |
| | | Scrt1 | (-/-) | scratch homolog 1, zinc finger protein (Drosophila) |
| | | Fbx16 | (-/-) | F-box and leucine-rich repeat protein 6 |
| | | Gpr172b | (-/+) | G protein-coupled receptor 172B |
| | | Adck5 | (-/+) | aarF domain containing kinase 5 |
| | | Cpsf1 | (-/-) | cleavage and polyadenylation specific factor 1 |
| | | Slc39a4 | (-/-) | solute carrier family 39 (zinc transporter), member 4 |
| | | Vps28 | (-/-) | vacuolar protein sorting 28 (yeast) |
| | | Mfkbil2 | (-/-) | nuclear factor of k light peptide gene enhancer in B cells inhibitor-like 2 |
| | | Cyhr1 | (-/-) | cysteine and histidine rich -1 |
| | | Kifc2 | (-/+) | kinesin family member C2 |
| | | Foxh1 | (-/-) | forkhead box H1 |
| 16 | MuERV-14 | Setd4 | (-/-) | SET domain containing 4 |
| | | Cbr1 | (-/+) | carbonyl reductase 1 |
| | | Cbr3 | (-/+) | carbonyl reductase 3 |
| | | Dopey2 | (-/+) | dopey family member 2 |
| 18 | MuERV-20 | Zfp236 | (+/-) | zinc finger protein 236 |
| 19 | MuERV-32 | Lgi1 | (+/+) | leucine-rich repeat LGI family, member 1 |
| | | Tmem20 | (+/+) | transmembrane protein 20 |
| | MuERV-9 | Sfxn4 | (+/-) | sideroflexin 4 |
| | | Prdx3 | (+/-) | peroxiredoxin 3 |
| | | Gprk5 | (+/+) | G protein-coupled receptor kinase 5 |
| | | | | |

Chr (Chromosome number), Ori (Orientation). Gray highlight indicates the annotated genes harboring an ERV in the intron. *MuLV-ERV newly identified in this study. Please refer to Table 3 for the MuLV-ERV names.
